# Unnecessary Operations on Women

**Published:** 1906-12

**Authors:** Lewis S. McMurtry

**Affiliations:** Louisville, Ky.


### "Unnecessary Operations on Women."

EDITOR SURGERY, GYNECOLOGY AND OBSTETRICS.

SIR: The last number (October) of your admirable journal contains a communication with the above title, by Dr. Arthur Dean Bevan, which casts undeserved reproach upon a class of honorable surgeons engaged in special practice. Such an indictment as is drawn therein should not pass before the attention of your many readers unchallenged. Every one who knows Dr. Bevan personally, as I do, knows that he is incapable of *intentionally* doing injustice to any individual or class of individuals, or to any good motive or principle. Hence, I believe that his observations have been faulty, or perhaps, unfortunate; for his deductions are unwarranted by the facts.

After tersely reciting the marvelous advances of modern surgery, which have resulted in rescuing thousands from otherwise incurable disease and relieving inestimable suffering, Dr. Bevan declares that with this great boon to humanity there has been developed *a curse*. which curse consists in the doing of operations which are unwarranted and unnecessary. He says the principal sufferers are women, who are prone to believe their sexual organs are diseased when consulting a physician, and ready to submit to operation, if advised. Further, he states, "the principal offenders have been men who have limited their work to gynecology;" that "there are many gynecologists who do not think of taking charge of a woman except for an operation, and the percentage of women who apply to them for advice, and in whom they find no indication for an operation, is so small as hardly to form, as the chemist would say, a trace in the sum total." He then asks the question: "Are all these operations necessary?" and thus answers it: "In reply to this question I shall say that my own impression of the surgical work done on women, especially that done by men who limit their work to gynecology, is that certainly thirty per cent. of it is unnecessary and unwarranted." In a word, Dr. Bevan declares that almost one third of the operations done by gynecologists are unnecessary and unwarranted. This is a most serious accusation; and, if true, would justly deprive a large class of special surgeons of the confidence and respect of their professional brethren and also of the general public.

I quite agree with Dr. Bevan that with the modern hospital and trained operating-room nurse, aseptic results are made easy; and, as a result, needless operations are done and operations are undertaken by those who have neither the knowledge nor training to fit them for such work. But I deny that special surgeons are given to this wrong more than general surgeons.

Unnecessary and unwarranted operations can only obtain from two causes: imperfect knowledge and skill, or dishonesty, on the part of the surgeon who advises and does such operations. Now, is it true that gynecological surgeons are unequal in scientific knowledge and skill to their confreres engaged in other departments of surgery; or are they less honest than other practitioners? Dr. Bevan says "the same criticisms are to be made of the general surgeon or the operator in any specialty who, for fee or through ignorance or misguided enthusiasm, submits a human being to the risks and costs of an unnecessary or unwarranted operation;" and again: "I have singled out the gynecologist because he is responsible for much of this work." It will be noted that our author leaves no one in doubt as to the particular body of practitioners against whom his accusation is directed.

Now, is it true that gynecologists are deficient in scientific knowledge or surgical skill? On the contrary, any account of the great advances and achievements of modern surgery would be glaringly inaccurate did it not concede to gynecologists a most conspicuous place both in its foundation and development. Who, in the development of abdominal surgery, which is the crowning glory of modern surgical achievement, did more to blaze the way than Marion Sims? He first, after the discovery of antiseptic methods of operating, directed attention to the surgery of the gall-bladder and ducts, and advocated immediate operation for penetrating wounds of the abdomen. Who, after Lord Lister, did more to simplify and perfect the modern operative technic, and, at the same time, to extend the scope of intraperitoneal surgery than Lawson Tait? These two men are the founders of modern gynecology. And have their successors and followers been wanting in scientific knowledge or operative skill? Have the numerous men engaged in teaching and practising gynecology in more recent years, proved unworthy of their predecessors? It is well known to the profession that pelvic surgery in women has attained a degree of refinement and precision unsurpassed in any field of surgery, and that gynecological surgeons have borne a most conspicuous part in developing the surgery of the gallducts and liver; also of appendicitis, and of the urinary tract. In a word, modern abdominal surgery is, in great part, an outgrowth of pelvic surgery, and gynecological surgeons have been foremost in this great achievement. Certainly Dr. Bevan cannot have these men in mind when he describes some gynecologists as having "so limited their vision to their specialty that they believe that it and they are the center of the pathological female human universe about which all else revolves."

The principal feature of Dr. Bevan's article to which my protest is directed is that he has singled out a special class of the profession to which he distinctly attributes an exceptional proportion of dishonesty and wrong-doing. This is altogether without any warrant. Gynecological surgeons under every test stand the peers of any other class of physicians or surgeons.

In the development of new fields of surgical work some errors are unavoidable. I allude to the errors that are corrected by increasing experience and accumulating knowledge. Such errors have occurred both in special and general surgery.. As apt illustrations I might cite the unnecessary removal of the ovaries which followed the pioneer work of Battey, Hegar and Tait; and the excision of the coccyx for neurasthenic tenderness, for some time practised by general surgeons. In the correction of these errors, the gynecologists were foremost in stopping abuse and establishing correct standards of practice. This is attested by the extensive space devoted to conservatism in both the periodical and textbook literature of modern gynecology. I am sure of my ground when I say that in the practice of gynecologists, judging from my personal knowledge of their work, in quite one third of the patients who are referred, or apply, for operation, operation is *not* done. It is my knowledge upon this point which impels me to resent Dr. Bevan's charge that thirty per cent. of the operations done by gynecologists are unwarranted and unnecessary. Can the same discrimination in deciding the question of operation be accredited the general surgeon in gynecological cases as is exercised in those cases by the gynecologist? Does concentrated study and exceptional experience in a special field of research and practice impair surgical judgment and lessen one's resources in treating that special class of cases? On the contrary, it is well known that error is more common with those of desultory and superficial study, with lessened clinical experience. Surely, no one would claim that the moral responsibility of a surgeon is affected by either special or general surgery. That is a condition inherent in the individual, and is not altered by any particular field of professional labor.

In conclusion, I must dissent wholly from Dr. Bevan's estimate as to specialism in medicine and surgery. Pervading his entire article is a depreciation of specialism in every department of surgery. If he would protest against specialism without broad general knwledge of pathology and training in general medicine and surgery, his position could not be questioned. But no one can belittle the great offices of special work—like neurology and pediatrics in medicine, or such as ophthalmology, gynecology and orthopedics in surgery—in advancing scientific knowledge and perfecting the results of treatment.

The late Samuel D. Gross, the foremost surgeon of his day, died during the first decade of the antiseptic era. With clear vision he realized that the golden age of surgery was at hand. After recounting the advances established and assured by Lister's great discovery, he wrote of the influences so potent in advancing surgery, as follows: "Specialism," too, is entitled to undying praise. It has penetrated with its methods and instruments of research the innermost recesses of the human body, and has achieved, in a comparatively brief period, triumphs which general surgery could not have achieved in half a century, if ever." I submit that these words are as applicable to the surgical work of the present time as when penned by the Nestor of American Surgery.

(*Signed*) LEWIS S. MCMURTRY.

LOUISVILLE, KY., NOVEMBER 1, 1906.

---

1. NOTE.—The foregoing correspondence appeared in *Surgery, Gynecology and Obstetrics*,—Dr. Bevan's letter in October, and Dr. McMurtry's in November. It is reproduced here because of its importance, and to give it to such of our readers who may not chance to see it in the original. We make editorial reference elsewhere to this remarkable correspondence.—Editor BUFFALO MEDICAL JOURNAL.

